# Progress in Medicine

**Published:** 1898-04-23

**Authors:** 


### Progress in Medicine.

### RHEUMATISM AND GOUT.

The constant recurrence of a reported discovery of the microbe peculiar to rheumatism has been a well-marked feature of medical literature of late years. Pierre Achalme[1] describes in the "Annales de l'Institut Pasteur" an anærobic microbe which he has found in nine cases of acute rheumatism. The bacillus is a large rod, like that of anthrax, forming lengthy filaments, is slightly motile when young, and stains with the ordinary anilin dyes, and is not discoloured by Gram's method. If grown in milk coagulation and a great evolution of gas take place. Inoculated into animals, it produces dilatation of the arterioles, and thrombosis and negative chemiotaxis. Its development in cultures is hindered by the addition of sodium salicylates, and heart lesions resembling those of human beings can be produced by it in rabbits. On the question of the pathology of rheumatic endocarditis in men, Singer[2] finds that it generally follows, and does not precede, the joint lesions, and it results from various organisms in the blood, most often from the pyogenic cocci, which the writer is inclined to look on as the actual cause of rheumatism. Either endocarditis or rheumatism may follow an attack of tonsillitis. A case illustrating the favouring influence of various bacterial poisons on rheumatism is given by W. C. Dickinson.[3] The patient first suffered from supposed influenza; afterwards acute rheumatism with joint affections came on, and was relieved by salol. During convalescence the patient caught diphtheria, for which anti-toxin was given with marked benefit. Scarcely had the temperature fallen when a relapse of rheumatism with peri- and endo- carditis took place. Pneumonia, pleurisy, and effusion followed, but the patient gradually recovered from all these troubles, and made an excellent convalescence. The diphtheria was verified by cultures, and the question arises whether the diphtheria or the anti-toxin can be held to exercise a definitely favouring influence on the rheumatic virus. An interesting discussion took place at the Clinical Society upon sudden death in acute rheumatism, when Herringham[4] gave the history of an occurrence of the kind. The joint pains had quickly yielded to salicylates, but the patient complained of epigastric and precordial pain, and became pale and cyanosed. Sudden death followed, when besides œdema of the lungs, fatty degeneration of the walls of both ventricles and proliferation of cells in the interstitial connective tissues were discovered. Thus an acute myocarditis would seem to be one of the causes of sudden death in this disease. Maclagan added that delirium, when there was no hyperpyrexia or pneumonia present, was usually a symptom of this myocarditis, of which the existence of precordial pain and cyanosis were also important indications. Duckworth laid stress on the presence of two additional signs—loss of tone of the heart sounds, especially of the first one, and fall of the pulse tension. He agreed that sudden death was not improbable at times in these cases.

Besides Achalme we may here refer to another author, Riva,[5] who has made researches into the cause of the rheumatic toxin. With minute precautions he took fluid from the knee-joints of acute cases and inoculated it into a culture medium obtained from the synovial fluid of horses. He found a growth from every case on this medium, while that on ordinary culture tubes failed. The microbe seems to be an egg-shaped body; but this disappeared later on, and was followed by two kinds of bacilli. The same growth was obtained in all the eight cases. Acute myositis has been found as a sequel of rheumatism by H. Risse.[6] There was swelling of all the muscles of one leg, with œdema of the skin. The muscles of the other leg and abdomen were next invaded, and the attack lasted two months. Some atrophy of the affected muscles, but considerable power was found after the acute stage was passed. G. F. Still[7] draws attention to the difficulties of diagnosis of rheumatism in childhood, remarking that, out of 50 cases of it admitted at Great Ormond Street, 37 had signs of endocarditis, 23 showed rheumatic nodules, while, on the other hand, the family history is deceptive, and articular swellings about the joints of infants are almost always not rheumatism. The disease, indeed, is very rare in infants, and the symptoms are either from scurvy, syphilitic, and other epiphysitis, the acute arthritis of infants, gonorrhœal arthritis, and even from infantile paralysis. However, the common rheumatism of the hip-joint in older children is frequently taken for morbus coxi, and even for appendicitis, and may appear in two forms, the acute transient one and the chronic fibrous type, the latter causing thickening and immobility. The rheumatoid arthritis, too, of children may appear in two ways. The first type, which commences often in the first three years of life, attacks nearly all the joints at once, forming fusiform thickenings without change or outgrowth of the bones, and also without heart affections or nodules under the skin, but the spleen and other glands are frequently enlarged. The other form occurs later in life, generally after the sixth year. There are outgrowths of bone, and in later stages grating of the denuded joints, but here again there are no heart complications, and, indeed, the spleen and glands are also unaffected.

A different type of arthritis, but difficult to distinguish at times from rheumatism, is seen following acute diseases, scarlatina, diphtheria, typhoid, and influenza. In posterior basic meningitis an arthritis sometimes appears, due to the diplococcus, which does not, however, seem to go on to suppuration, as do some of the last mentioned cases. Septic processes in the lung and elsewhere also present forms of arthritis, which need to be distinguished. Of course, too, tubercular disease of the joints must not be overlooked in diagnosis. An arthritis occasioned by the pneumococcus is sometimes seen in adults, and was recently described by Fernet.[8] The sterno-clavicular joint was attacked in his two cases. There was pain, swelling, and peri-articular œdema, followed by grating. The joints yielded no pus, but the ligaments and cartilages were entirely destroyed, and a pure cultivation of the microbe was found in them. An extraordinary instance of arthritis in hæmophilia is recorded by T. E. Shaw.[9] Attacks of swelling had occurred in the knees at various times in the life of the patient, who had attained the age of 30, and suffered from hæmophilia. On one of these occasions blood was aspirated from the joint. Bony enlargements were present at the elbows and knees, with grating and outgrowths, but the hands and feet were not affected as in rheumatoid arthritis. Besides the permanent enlargements the joints were subject to frequent attacks of effusion, preceded by a feeling of heat and pricking, and passing off in two days. There were also periods of renal colic, after which clots of blood appeared in the urine. These joint lesions in hæmophilia are very imperfectly described in recent literature, but references are found in the St. Bartholomew's Reports, and in the *Medical Record* of 1896, to cases where resection was followed by fatal bleeding.

[1] N. Y. Med. Jour., Feb. 12. [2] Brit. Med. Jour., Dec. 11. [3] Lancet, Jan. 15. [4] Brit. Med. Jour., Feb. 5. [5] Brit. Med. Jour., Feb. 19. [6] Inter. Med. Mag., Dec. [7] Clinical Jour., Mar. 23. [8] Lancet, Dec. 11. [9] Lancet, April 2.

### DISEASES OF THE HEART AND CIRCULATION.

**Physiology.**—W. T. Porter[1] has recently made important experiments on the influence of the coronary circulation in maintaining the function of the heart. The experiments were done on dogs, and consisted in ligaturing or plugging the coronary arteries. It was found that the frequency of arrest of the heart is in proportion to the size of the artery ligatured; thus ligature of the circumflex branch causes arrest in 64 per cent. of animals, whilst no arrest occurred after ligature of the septal artery. The injury of the tissues surrounding the coronary arteries does not account for the arrest; the phenomena are due to the sudden stoppage of the nutrition of a considerable part of the heart.

It was long ago shown by Gaskell that the sequence of contraction of the various chambers of the heart is due to a rhythmic peristaltic wave of contraction spreading along the muscular fibres from the venous to the arterial end, and that the ganglion nerve cells have nothing to do with the matter. Gaskell proved this in the case of fishes, amphibia, and reptiles, but not in mammals. The difficulties in experimenting with the isolated mammalian heart have been ingeniously overcome by Porter,[2] who, having isolated a dog's heart, tied a cannula in the ramus descendens of the left coronary artery, and then cut away all the auricles and ventricles except that portion supplied by the descendens itself, which was fed by a stream of warm saline mixed with dog's blood. The portion of ventricle soon began to beat with great vigour, and did so for a long time. It follows that the cause of the rhythmic contraction lies in the ventricle itself; and that the integrity of the whole ventricle is not essential. Similar experiments were done with only a bit of the ventricle near the apex—which is devoid of nerve cells—showing that Gaskell's view in its entirety is true also of the mammalian heart.

Magrath and Kennedy[3] have made experiments on the relation of the volume of the coronary circulation to ventricular contraction in the isolated heart of the cat. They find that the force of the ventricular contraction is immediately affected by a change in the amount of blood supplied to the cardiac muscle, the ventricle beating with more force if the volume of the coronary circulation is increased, and with less force if diminished. The frequency of the ventricular contraction, on the contrary, is largely independent of the blood supply to the cardiac muscle, and may remain almost unchanged, whilst the force of contraction, in consequence of lessening the supply of blood, is reduced.

Howell[4] has recently published some valuable plethysmographic tracings showing the changes in the volume of the arm during the whole period of a night's sleep. The tracings show that the arm expanded as sleep supervened, reached a maximum of expansion in the course of the first hour or so, and remained expanded during the whole period of sound sleep. It was only as the waking state approached that the constriction of the limb again became evident. These experiments prove that during sleep there is a determination of blood to the limbs, and taken in association with those of Hughlings, Jackson, Hutchinson, and many others, support the conclusion that sleep is associated with a condition of cerebral anæmia. The further question is whether it is due to this cerebral anæmia. According to the theory that it is, sleep is due primarily to exhaustion of the vasomotor centre, fatigued by the constant instreaming of sensory impulses during the day; this leads to relaxation of the tone of the blood vessels, the brain hence becomes anæmic, and sleep ensues.

Hill[5] has, however, been led to doubt whether this is the sequence of events. Experiments were made on healthy children and young adults with the Hill-Barnard sphygmometer. This instrument consists of a leather armlet inside which is fastened a flaccid rubber bag, a force pump provided with an escape valve, and a pressure gauge graduated in millimetres of mercury. The armlet is put round the upper arm, and the pump is worked, raising the pressure within the rubber bag and gauge until the point is reached where the index gives the maximal cardiac pulsation. This point indicates the mean arterial tension, for when the mean pressure without and within the brachial artery is the same, the wall of the artery is at the systole and diastole able to oscillate with the greatest freedom. By means of this instrument Hill found that the mean arterial pressure falls considerably as sleep comes on, *e.g.*, in one person from 120 to 90 m.m. of mercury. This at first sight seemed to confirm the view that sleep is due to cerebral anæmia; but on further examination it became evident that the fall of arterial pressure is concomitant with sleep rather than the cause of sleep. A fall of pressure is invariably associated with warmth and rest in the horizontal position; moreover, when lying awake in the morning the fall of pressure is as great as when lying sleepy at night. Hence it is not possible to ascribe the causation of sleep directly to the fall of pressure.

The expansion of the limbs during rest and sleep is due to several causes: (1) the compressive action of the muscles ceases, (2) the respiration slows, and (3) the rate of the heart lessens owing to the cessation of external stimuli, the condition of warmth, and the horizontal position. At the same time the vaso-motor tone of the arteries lessens. Hill concludes that the anæmia of the brain is caused by rest of the body and the cessation of powerful objective and subjective stimuli, and it is the cessation of the latter which produces sleep. It would be as reasonable to ascribe sleep to the fall of temperature or diminution in the output of $Co_2$, as to the diminution of arterial pressure; all three, with other phenomena, are concomitants, not the causes, of sleep, and the ultimate cause of this we are still ignorant of.

[1] W. T. Porter, Jour. of Exper. Med., Vol. I., p. 49. [2] W. T. Porter, Jour. of Boston Soc. of Med. Sciences, March, 1897. [3] Magrath and Kennedy, Jour. of Exper. Med., Vol. II. [4] Howell, Jour. of Exper. Med., Vol. II. [5] Hill, Lancet, Jan. 29, 1898.

